# Surface Damaging of Brass and Steel Pins when Sliding over Nitrided Samples Cut by Finishing and Roughing EDM Conditions

**DOI:** 10.3390/ma13143199

**Published:** 2020-07-17

**Authors:** Vitaliy Martynenko, Daniel Martínez Krahmer, Amelia Nápoles Alberro, Amado Cabo, Daniela Pérez, Enrique E. Zayas Figueras, Hernán A. Gonzalez Rojas, Antonio J. Sánchez Egea

**Affiliations:** 1 Center for Research and Development in Mechanics, National Institute of Industrial Technology (INTI), Avenida General Paz 5445, Buenos Aires 1650, Argentina; vmart@inti.gob.ar (V.M.); dmartinez@inti.gob.ar (D.M.K.); danielap@inti.gob.ar (D.P.); 2 Faculty of Engineering, Universidad Nacional de Lomas de Zamora, Juan XXIII y Camino de Cintura, Buenos Aires 1832, Argentina; 3 Department of Mechanical Engineering, Universitat Politècnica de Catalunya, C. Jordi Girona, 1–3, 08034 Barcelona, Spain; amelia.napoles@upc.edu (A.N.A.); enrique.zayas@upc.edu (E.E.Z.F.); hernan.gonzalez@upc.edu (H.A.G.R.); 4 IONAR S.A. Avenida Arias, 342, C1430CRB Ciudad Autónoma de Buenos Aires, Argentina; cabo@ionar.com.ar; 5 Department of Mechanical and Metallurgical Engineering, Pontificia Universidad Católica de Chile, Av. Vicuña Mackenna 4860, Región Metropolitana 7820436, Chile

**Keywords:** plasma nitriding, EDM, pin-on disc test, surface quality, wear and friction

## Abstract

In the forging industry, surface quality and surface treatments of dies are crucial parameters to extend their life. These components are usually machined by milling or by Electrical Discharge Machining (EDM), and the final surface roughness depends on the machining techniques and operational conditions used in its fabrication. After milling, a nitriding treatment is widely applied to extend its service life. Nevertheless, no scientific report that informs about nitriding after EDM has been found. Accordingly, this work focuses on the wear and friction behavior of pins made of brass and medium carbon steel sliding over AISI H13 discs, made by wire EDM in the conditions of finishing and roughing. The discs are plasma nitride, and their effect on the wear during pin-on-disc tests is evaluated. In this sense, the analysis of the surface damage for the different pins will help us to understand the service life and wear evolution of the forging dies. The results show that plasma nitride reduces the friction and prevents the degradation of the pin, independently of the material of the pin, when sliding over finishing and roughing EDM conditions.

## 1. Introduction

Forging is a metal forming process widely used in industries, such as automotive, aerospace, railway, naval, oil, mining, and health [1,2]. Several parts are involved in this process: press, dies, material to be forged, lubrication system, and type of lubricant. Press and dies have to bear fatigue stresses, and dies are commonly manufactured with H series tool steel, where AISI H13 grade is the most universally used. This tool steel is hard to machine because of the high cutting tension due to its alloy content [3]. Replacing dies represent between 10% and 30% of the cost of a forged part [4], which enforces the need to decrease this percentage. In that sense, a proper forging process must take into account how the die is manufactured and during the forging process, protect and lubricate.

The die’s surface quality is relevant in the forging process to manufacture the forged part without surface damage or scratches. Accordingly, different options exist to manufacture dies, and depending on the option, different surface features can be achieved. One option is milling the dies. Here, parameters such as type of tool and its geometry [5,6,7,8], the machining strategy [9,10] and the operational conditions [11,12] play an essential role on its final surface properties, and these parameters are tightly related to the service efficiency of the die (for example, fatigue resistance). Another option is the Electrical Discharge Machining (EDM) of the dies [13]. EDM is capable of making complex shapes and deep cavities; the only requirement is to work with an electrically conductive material. The necessary conditions to take into account in the EDM process that can affect the EDM removal rates and surface finishing of dies are the following: the material of the electrodes [14], the frequency of pulses and depths [15] and the addition of external powders [16] to modify surface properties and minimize the roughness by filling pores and cracks. Once the dies are manufactured, different surface treatments are used to extend their service life [17]; the most used treatment is the diffusion of nitrogen (nitriding), which achieves a significant increase of surface hardness and increase up to 125% in fatigue life [18]. The plasma nitriding is a modern technology that depends mainly on: gas composition, voltage and duty cycle, pressure, time, and temperature. For example, Solis-Romero et al. [19] used pin-on-disk tests at room temperature with several axial loads. In all cases, the friction coefficients were reduced for nitrided samples. A similar nitriding procedure was performed by Leite et al. [20] at 400 °C for 4 h, 9 h, 16 h, and 36 h. Then, during the ball-on disk test, a decrease in wear rate was found by up to 50% when the nitriding time passed from 4 to 36 h. Other experiments were made, modifying the internal pressure at the plasma nitriding chamber [21]. These authors found that the friction coefficients went from 0.55 to 0.30 for nitrided samples with pressures of 200 to 300 Pa, respectively.

Besides the technology of manufacturing the dies and the type of their surface treatment, it is necessary to consider the medium or lubricant used during the forging operation. A graphite-based lubricant is commonly used due to the high temperature reached in hot and warm forging processes. Dual-phase lubricants, spreading over the surface, reduce the friction coefficient during the forging process. Previous works [22,23,24] analyzed the behavior of several commercial graphite-based lubricants in hot forging, employing wear tests like pin-on-disc and friction tests like ring compression test, performed in different surface conditions. The experience shows that the size of the embedded graphite particles, the graphite concentration, and the lubricant’s kinematic viscosity are parameters that influence the friction coefficient at the interface, independent of the testing temperature. This work aims to study the initial surface properties to describe their influence on surface wear. These surface properties of the discs are defined by wiring EDM in finishing and roughing conditions and using plasma nitride. Then pin-on-disc tests are performed to compare the friction coefficient and wear rate for pins made of brass and medium carbon steel, which slide over discs made of AISI H13.

## 2. Methodology

This section is divided into several subchapters: characteristics of the lubricant, machining conditions and surface treatment of the discs, and pin-on-disc tests at room temperature. Throughout these subsections, the experimental protocols, equipment, and facilities used to investigate the friction and wear of steel and brass pins on EDMed discs with different types of surfaces conditions are described.

### 2.1. Characteristics of the Lubricant

**Figure 1:**
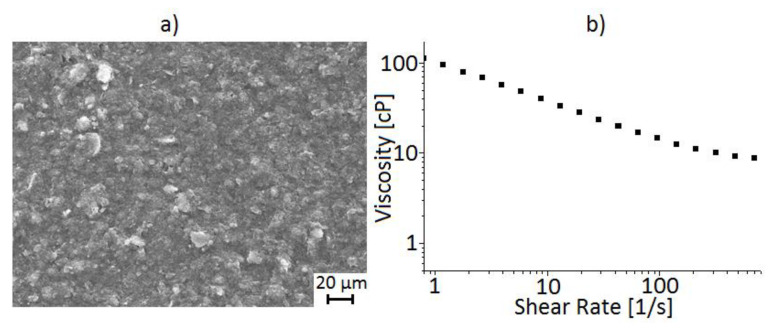
(**a**) Graphite-based lubricant matrix and (**b**) the kinematic viscosity curve of the water diluted lubricant.

The graphite-based lubricant was diluted up to 5% in water, which corresponds to a typical average lubricant used by Argentine forging companies. This lubricant had a density between 1.10 and 1.20 g/cm^3^. A scanning electron microscopy (FEI Model: QUANTA 250 FEG, FEI, Eindhoven, the Netherlands) was used to determine the elemental chemical composition of the lubricant and the size of the graphite particles. Also, the kinematic viscosity was determined with an oscillating rheometer (Anton Paar Physica Model MCR301, Anton Paar, Ostfildern, Stuttgart, Germany). Figure 1a shows the as-received graphite lubricant matrix as supplied, and Figure 1b presents the kinematic viscosity curve for the water diluted lubricant. Table 1 quantifies the average size of the graphite particles, which are obtained from 20 measurements. Also, the average percentage of the weight of each chemical element corresponds to the result of five measurements.

### 2.2. Discs and Pins Preparation

A Promecor SMT 19/500 numerical control lathe (Promecor, Córdoba, Argentina) was used to machine 16 AISI H13 steel discs with an external diameter of 63 mm, an internal diameter of 19 mm, and 8 mm of thickness. All discs were hardened at 1040 °C and tempered for 2 h at 570 °C. Both faces of the discs were grounded on a tangential grinding machine (Davonis model SGS-1230AHR, Davonis, Billinghurst, Buenos Aires, Argentina), using an A46I10V grinding wheel with an average grain size of 0.38 mm. A hardness tester (INSTRON WOLPERT, Model: S8-233971, Rockwell C Scale, Instron Corporation, Canton, MA, USA) was used to measure the hardness of the disc before the wire EDM and nitriding treatment, being of 51.6 ± 1.5 HRC for finishing condition and 51.1 ± 2.1 HRC for roughing condition. Finally, the surfaces of the discs were wire EDM (Novick AR 35 MA) using two operational conditions: half of them in a finishing condition (t_on_ = 5 μs; t_off_ = 34 μs; T = 39 μs; f = 25.6 Hz; t_on_/T = 0.13; V = 4 V), and the other half in a roughing condition (t_on_ = 50 μs; t_off_ = 180 μs; T = 230 μs; f = 4.3 Hz; t_on_/T = 0.22; V = 6 V). From these two groups, half of the discs were plasma nitriding by IONAR S.A. The nitriding conditions were: temperatures of 500 °C, exposure time of 13 h, protective gas made of 20% N_2_ + 80% H_2_, and a pressure of 6 hPa. Finally, a transversal section of the nitride combined with finishing and roughing EDM conditions was analyzed in a Vickers microhardness tester (Shimadzu HMV-2000, Shimadzu Corporation, Kyoto, Japan). Regarding the pins, they come from drawn bars of 6.35 mm in diameter. Sixteen (16) pins were made of brass ASTM B16 hardness HRB 76, and another 16 pins were made of steel AISI 1045 hardness HRB 87. All of them were made with hemispherical tips 4 mm diameter at both ends that were polished with abrasive papers of grain sizes 100, 600, and 1000.

### 2.3. Pin-on Disk Test at Room Temperature

**Figure 2:**
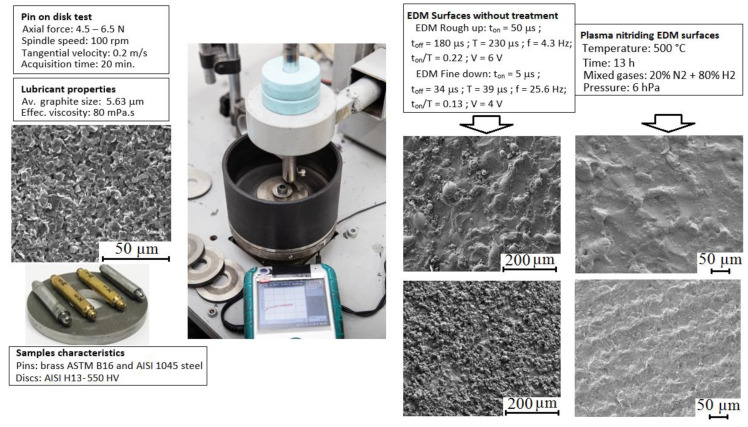
Pin-on-disk equipment used in this work and data for different experiments.

The pin-on-discs tests were carried out with equipment made in-house (Figure 2) at INTI-Mechanical Center in Argentina. The tangential force during the tests was recorded with a data logger (Vernier Model LabQuest, Vernier, Beaverton, OR, USA), which has a load range up to 50 N. Pins and disks were fixed in the machine to carry out the tests with the appropriate amount of lubricant. The lubricant was added on the disc’s surface, checking that a uniform dispersion had occurred before the test began. The axial loads on the pins were 4.5 N for the brass and 6.5 N for the steel, to reproduce a contact pressure similar to that which occurs in a forging process with these materials (140 and 200 MPa respectively [25]). The tangential speed in the pin-disk contact was 0.2 m/s, and the total experiment duration of each test was 20 min (equivalent to 240 m). These experiments were repeated 32 times resulting from the combination of two-pin materials, two discs machining conditions (finishing and roughing wire EDM), two surface treatment conditions (non-nitrided and nitrided discs), and four repetitions per each condition. From the experiments, friction curves and weight loss of the pins were determined. The weight difference was measured with a Radwag electronic scale, model AS 220.R2 (Radwag, Radom, Poland), with an accuracy of 0.1 mg. Finally, the adhesions of material on discs were studied to describe the surface properties. The pin-on-disc tests were performed at 21 ± 3 °C and relative humidity of 50 ± 10%.

### 2.4. Surface Roughness

As the EDM process does not have a predominant surface roughness direction [26], the surface roughness measurements were made in random (multidirectional) directions. A portable roughness meter (Taylor Hobson Surtronic 3+, Taylor Hobson, Leicester, England) measured the arithmetic mean surface roughness (Ra) and the total height of the roughness profile (Rt), where the cut-off and the evaluation length were set at 0.8 mm and 4 mm respectively. The AISI H13 steel discs were turned quenched, tempered and finally grounded up to a Ra of 0.51 ± 0.08 µm. Table 2 shows average values and the standard deviation of the surface roughness of the discs measured on the surfaces obtained from finishing and roughing EDM with and without the plasma nitriding treatment. A total of 96 measurements were made to determine arithmetic mean surface roughness (R_a_) and the total height of the roughness profile (R_t_).

**Figure 3:**
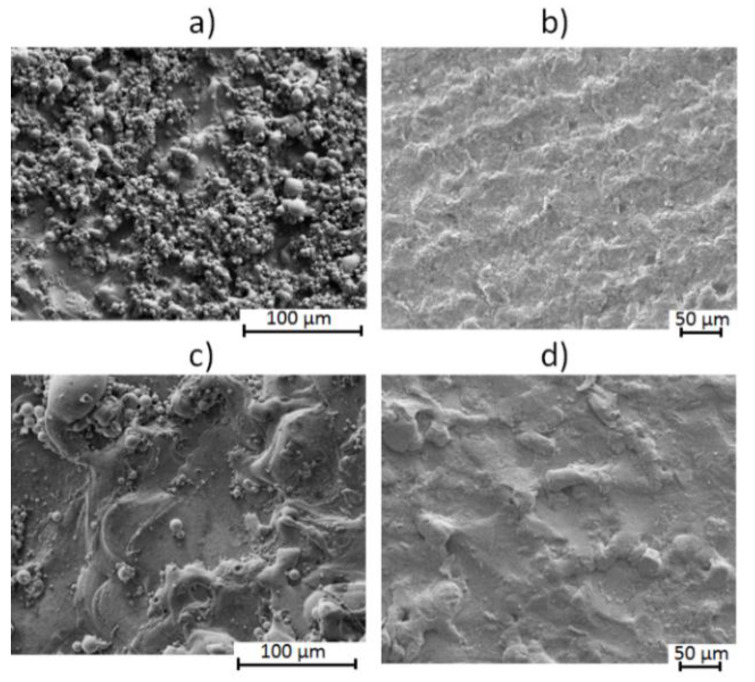
(**a**) Surface morphology of EDM in roughing condition; (**b**) Surface morphology of EDM in roughing condition and nitriding; (**c**) Surface morphology of EDM in finishing condition; (**d**) Surface morphology of EDM in finishing condition and nitriding.

The finishing and roughing EDM conditions have significant differences in the surface roughness, being higher for the roughing condition of EDM. The nitrided specimens presented lower values of Ra and Rt, as well as lower dispersion. Similar trends were also found by Solis Romero et al. [19]. Figure 3 shows the surface morphologies for finishing and roughing EDM surfaces without and with the plasma nitriding treatment. Regarding the material’s hardness, the specimen treated in a finishing EDM condition presented a hardness 32.5% higher than the roughing EDM condition. Nitrided finishing EDM specimens reached similar hardness values to [18]. Regarding the EDM surface morphology (Figure 3a,c), the results present the same features as the work reported in [13], where the sample was EDMed with short pulses and a long duration.

## 3. Results and Discussion

This section analyzes the friction curves to describe the influence of the surface roughness and the nitriding treatment on the friction coefficients. Besides, the weight loss of the pins after the pin-on-disk tests is analyzed to measure the wear of tool tips for the pins made of brass and steel.

### 3.1. Pin-on Disk Tests

**Figure 4:**
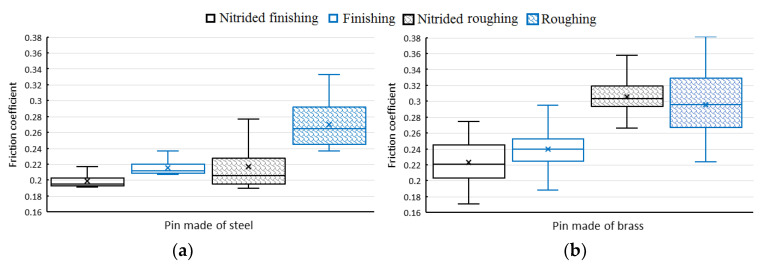
Boxplots of the friction coefficients at the stationary phase for the different surface conditions with and without the nitriding treatment for (**a**) pins made of steel and (**b**) pins made of brass.

The experimental tests are repeated five times per each surface condition to analyze the friction coefficient at the stationary phase. Figure 4 exhibits the boxplot of the friction coefficients for the two surface conditions and with the presence or not of the nitriding treatment.

Regarding the pins made of brass, the friction coefficient shows no significant changes with and without the plasma nitriding treatment. However, as expected, the lower friction coefficient corresponds to the specimens processed by finishing EDM compared to roughing EDM. On the contrary, the friction coefficient of pins made of steel is noticeably reduced when the surface is nitrided, in particular, 7.6% for the finishing EDM and 19.4% for roughing EDM. In this sense, Solis Romero et al. [19] using pins made of AISI 52,100 on AISI H13 discs polished with silicon carbide emery papers and diamond paste to obtain mirror finish, found a decrease of 33% of friction coefficient when they treated the discs with plasma nitriding. Consequently, the lowest friction coefficient resulted in an average of 0.22, for both types of materials, with and without nitriding in finishing EDM condition. As a reference, similar friction values were found by Leite et al. [20], when testing Si_3_N_4_ balls on polished surfaces of nitrided discs made of AISI H13 steel.

### 3.2. Weight Loss of the Pins

**Figure 5:**
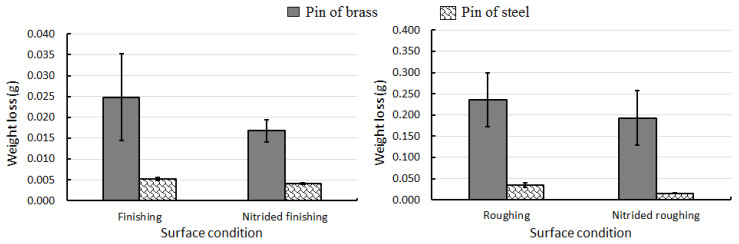
Weight loss of the pins made of brass and steel after the pin-on-discs test in the following surface conditions: finishing (**left**) and roughing (**right**) EDM conditions with and without nitriding.

During the pin-on-disc test, the pin is expected to wear because the material of the disc is harder than the pin. Accordingly, Figure 5 exhibits the weight loss of the different pins and surface conditions after the pin-on-disc tests. A cleaning procedure of 15 min of ultrasonic bath with isopropanol was performed in each sample before measuring the weight loss.

The weight loss values show that the average weight loss is about 1.22 to 2.32 times higher for brass and steel when comparing non-nitrided discs versus nitrided ones. The lowest surface degradation was found for a nitrided and roughing EDM condition, whereas the worst surface was denoted for non-nitrided and finishing EDM conditions. Das et al. [27] reported wear tests of non-nitrided and nitrided AISI H13 discs prepared by standard metallographic methods, and pins made of alumina with a semi-sphere morphology. They found that the weight loss was about three times higher for non-nitrided discs. Also, Sarkar et al. [28] performed pin-on-disc tests with pins of brass over discs of steel. They found a comparable weight loss to our results. In particular, they describe weight loss values lower than 100 mg for a traveling distance of approximately 150 m (240 m in this work). Table 3 shows the average weight loss and its standard deviation in increasing order for the different surface conditions (finishing or roughing) and treatments (with and without nitriding).

### 3.3. Surface Damage of the Tool Tip

**Figure 6:**
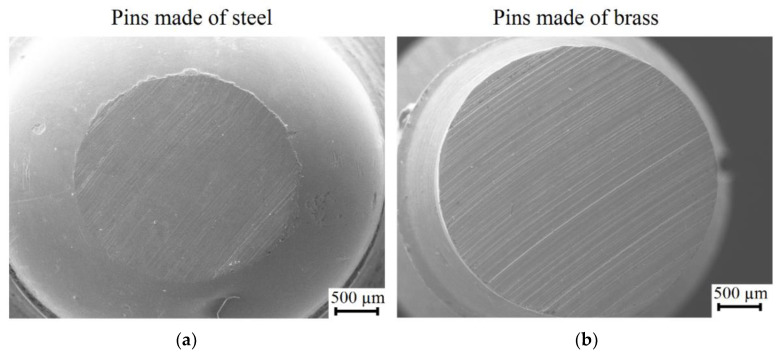
Pin tips after sliding on nitrided discs made by EDM with finishing condition. (**a**) Surface damage of a pin made of steel; (**b**) Surface damage of a pin made of brass.

As expected, the pins made of brass show higher tip degradation compared to the steel pins. Besides, the surface roughness presents a different influence on the type of material. A smooth surface favors the degradation of the brass pin, while lower degradation is found for the steel pin. The contrary happens when the coarse surface roughness is present on the surface of the discs. Figure 6 exhibits the SEM images of the tips after sliding (pin-on-disc test) over nitride discs with a finishing EDM process. The results show that the lowest weight loss was found for pins made of steel with the finishing EDM process, sliding over nitrided discs. On the opposite, the highest weight loss was found for pins made of brass sliding over non-nitrided discs in the roughing EDM condition. It is noticeable how after 20 min of sliding testing, the hemispherical morphology of the tips made of brass are wholly removed.

Finally, an EDS analysis (Energy Dispersive X-rays Spectroscopy) was performed on the discs to determine if material adhesion was on its surface during the sliding tests. Table 4 shows the EDS values to estimate Cu and Zn on the surface of the discs tested with brass pins. The EDS analysis shows similar adhesion values when comparing finishing and roughing surfaces. However, a significant difference is observed when comparing the adhesion of Cu and Zn between nitrided and non-nitrided surfaces. Note that the amount of adhesion of copper-zinc significantly increased in the non-nitrided discs. This behavior can be justified by the compatibility chart of Rabinowicz et al. [29] to justify the adhesion of Cu and Zn on the discs of steel. Zn and Cu have soluble values between 0.1 and 1% and, consequently, have a high tendency to adhere to iron.

## 4. Conclusions

The present study shows a thorough comparison of the friction and wear capability between two different EDM surfaces (finished and roughened) with and without nitriding treatment. Accordingly, some conclusions can be drawn:

- Nitriding significantly reduced the roughness, considering the arithmetic mean surface roughness (R_a_) and the total height of the roughness profile (R_t_). The roughing EDM process showed an increase of the surface roughness of 2.1 times in respect to the finishing EDM process. The nitriding treatment decreases 31% of the surface roughness on average.
- The material hardness of the nitrided disc machined with a finishing EDM was 32.5% higher than for the same disc machined with a roughing EDM condition.
- With respect to the pin weight loss, nitrided discs reduce the pin degradation at least between 18.4% and 19.6% for brass and steel, respectively.
- The friction coefficient exhibits lower values for nitride finishing surfaces and higher values for non-nitrided roughing surfaces, independently of pin material. However, for brass pins, significant differences are found for the surface condition (finishing and roughing EDM), and no significant differences are denoted in the disc treated or not with plasma nitriding.

## Figures and Tables

**Table 1:** Chemical composition and particle size of the graphite particles.

Lubricant	C (wt.%)	O (wt.%)	Na (wt.%)	Al (wt.%)	Si (wt.%)	S (wt.%)	Graphite Size (μm)
Average	80.67	11.78	2.36	0.92	3.25	0.29	5.63 ± 2.27

**Table 2:** Surface roughness and material hardness for the different EDM conditions with and without nitriding.

Sample	R_a_ ± SD (μm ± SD)	R_t_ ± SD (μm ± SD)	Hardness (HV ± SD)
Finishing EDM (substrate)	3.74 ± 0.38	26.85 ± 3.39	556.0 ± 25.2 (*)
Roughing EDM (substrate)	7.88 ± 1.51	54.95 ± 8.93	543.1 ± 35.4 (*)
Nitrided finishing EDM	2.31 ± 0.40	18.09 ± 2.60	1130.3 ± 71.1
Nitrided roughing EDM	5.43 ± 0.76	43.07 ± 6.08	853.3 ± 26.4

* The substrate was hardened and tempered to record the hardness similar than [20].

**Table 3:** Average weight loss and its standard deviation in increasing order to identify which surface conditions and treatments present lower wear.

Configuration\Sample	Brass (mg ± SD)	Steel (mg ± SD)
Nitrided & finishing	1st	4.1 ± 0.2
Finishing	2nd	5.1 ± 0.3
Nitrided & roughing	3rd	15.0 ± 0.7
Nitrided & finishing	16.8 ± 2.7	4th
Finishing	24.8 ± 10.4	5th
Roughing	6th	34.8 ± 5.0
Nitrided & roughing	192.7 ± 64.4	7th
Roughing	236.1 ± 63.5	8th

**Table 4:** EDS values of Cu and Zn on the surface of the wore discs for the four type of surface conditions and brass pins.

Sample	Nitrided & Roughing	Roughing	Nitrided & Finishing	Finishing
Cu (wt.%)	0.38	1.46	0.28	2.02
Zn (wt.%)	-	0.89	0.22	1.10
